# Evaluation of Wound-Healing and Antioxidant Effects of *Marantodes pumilum* (Blume) Kuntze in an Excision Wound Model

**DOI:** 10.3390/molecules26010228

**Published:** 2021-01-05

**Authors:** Shihab Uddin Ahmad, Nor-Ashila Binti Aladdin, Jamia Azdina Jamal, Ahmad Nazrun Shuid, Isa Naina Mohamed

**Affiliations:** 1 Department of Pharmacology, Faculty of Medicine, Universiti Kebangsaan Malaysia, Jalan Yaacob Latif, Bandar Tun Razak, Cheras, Kuala Lumpur 56000, Malaysia; shihab@shsmu.edu.cn (S.U.A.); anazrun@yahoo.com (A.N.S.); 2 State Key Laboratory of Oncogenes and Related Genes, Renji-Med X Clinical Stem Cell Research Center, Ren Ji Hospital, Department of Urology, School of Medicine, Shanghai Jiao Tong University, Shanghai 200127, China; 3 Drug and Herbal Research Centre, Faculty of Pharmacy, Universiti Kebangsaan Malaysia, Jalan Raja Muda Abdul Aziz, Kuala Lumpur 50300, Malaysia; shila.aladdin@gmail.com (N.-A.B.A.); jamia@ukm.edu.my (J.A.J.); 4 Department of Pharmacology, Faculty of Medicine, Universiti Teknologi MARA, Sungai Buloh Campus, Jalan Hospital, Sungai Buloh 47000, Selangor, Malaysia

**Keywords:** *Marantodes pumilum*, wound healing, collagen, fibronectin, fibroblast, antioxidants

## Abstract

*Marantodes pumilum* (MP) is a great source of herbal medicine used traditionally by both men and women for various purposes. MP may have potential wound-healing effects due to its diverse biological properties. An extensive study was conducted in a normal male rat model for determining the effects of MP *var. pumila* (MPvp) and *var. alata* (MPva) on the wound healing process. Here, 126 male Sprague-Dawley rats were divided randomly into seven groups as follows: sham-operated (SH), vehicle dressing (VD), flavine dressing (FD), MPvp leaves (PL), MPvp roots (PR), MPva leaves (AL), and MPva roots (AR). The parameters studied were the percentage of wound contraction, histomorphology study by hematoxylin and eosin (H&E), Masson–Goldner trichrome (MGT), and immunohistochemistry (IHC) staining. In addition, the levels of enzymatic antioxidants and malondialdehyde were also measured in the wound tissue homogenates. Wounds treated with extracts (PL, PR, AL, and AR) showed significantly faster healing (*p* < 0.05) compared to untreated and control groups (SH, VD, and FD). Histological analysis among MP-treated groups revealed better re-epithelialization, higher collagen deposition, enhanced fibronectin content and fibroblast cells, and higher fiber transformation from collagen-III to collagen-I, accompanied with a significant surge in enzymatic antioxidant activities and a decline in lipid peroxidation. MP has antioxidant effects that may enhance wound healing in the rat model.

## 1. Introduction

Wounds are physical injuries associated with surgical procedures, falling, heat, infectious disease, or underlying pathological conditions of tissue that interrupt normal tissue functions [1]. Wound healing is a normal physiological process where a set of biomolecular events is involved. These biological processes are fundamental to restore the functional integrity of injured tissues associated with four sequential yet overlapping wound-healing phases; hemostasis, inflammation, proliferation, and remodeling [2]. At the start of the healing process, hemostasis, platelets are activated to the wound site and form coagulation by aggregating with fibrin protein to prevent further bleeding [3]. Immune cells release many inflammatory mediators that help to engulf pathogens and debris from the wound area during the inflammatory phase [4]. In addition, various growth factors are released to initiate the migration of cellular components toward the wound area. The proliferation phase is associated with many biochemical events, particularly new blood vessels’ formation by the vascularization of endothelial cells and granulation tissue formation due to deposition of extracellular matrix cross-linked with collagen, fibronectin, and fibroblast. Keratinocytes proliferate to form epithelialization and fibroblast differentiation into myofibroblast to reduce the wound size [5,6,7]. In the fourth phase, collagen becomes more mature and remodels along tension lines to become tough skin [8].

Wound healing is a complex process that can easily be interrupted by many factors such as infection, contamination, age, stress, oxygen, nutrition, medication, sex hormones, obesity, diabetes, and venous or arterial disease which lead to non-healing chronic wounds [9]. Therefore, factors that can delay the healing process of the skin should be considered during wound treatment. There are many medications available in the market to treat wounds. However, they have limitations, including high costs. Most wound-healing medications work as agents protected from infection but do not induce the healing process. Moreover, most of these medications have many side effects, especially when compared to natural products. Natural products are also preferred widely because of their potential as natural medicine and because they are easily obtainable from natural sources. Therefore, natural products are excellent potential agents for wound healing.

*Marantodes pumilum* (Blume) Kuntze (MP) is one of herbaceous plants in the Primulaceae family that contains lanceolate leaves with a creeping stem and long roots [10]. The plant is widely found in the South East Asian region, including Malaysia, Indonesia, Thailand, and China, with three common variants [11]. MP is known locally as “Kacip Fatimah” in Malaysia. It is one of the key plants in the Malaysian economy, funded to be developed for commercial purposes [12]. It is also recognized and highly demanded as traditional medicine by local Malay people because of its therapeutic effects. Numerous studies of MP have determined the bioactive phytochemicals including flavonoid and phenolic acids, methyl gallate, carotenoids, saponins, ascorbic acid, fatty acids, and benzoquinone compounds contributing to its therapeutic efficacy. Flavonoids such as apigenin, rutin, kaempferol, and myricetin and phenolic acids such as pyrogallol, gallic acid, and caffeic acid were found in MP [13,14,15,16,17]. Recent studies have been reported that MP has potential therapeutic effects to many diseases including metabolic disorders, osteoporosis, and cardiovascular diseases [18,19,20].

MP is to be believed to contain phytoestrogens because of its huge consumption by women in South East Asian communities for many health issues, particularly female reproductive-related problems [21]. A study [22] reported that both ethanolic and aqueous extracts of MP *var. alata* and *var. pumila* have phytoestrogenic properties. The aqueous extract of MP inhibited estradiol binding with the estrogen receptor of antibodies and suggested the existence of estrogen-like compounds [23]. In our previous study, the phytoestrogenic properties of MP were postulated to promote wound healing [24]. However, it was shown that there was no significant difference between the estrogen-treated and normal healing processes in an ovariectomized rat model. Therefore, phytoestrogens and estrogens may not have a key role in wound-healing processes as postulated at the beginning. Certain intrinsic properties or active compounds in MP can also be helpful to the wound healing cycle. Several ethnopharmacological and clinical studies demonstrated that MP accords many therapeutical effects including antimicrobial, antioxidative, and anti-inflammatory properties [25,26,27]. As wound injury is a common event for both men and women, this study was conducted to determine the effectiveness of MP aqueous extract in the form of topical application for the healing of skin wounds in a normal male rat model.

## 2. Materials and Methods

### 2.1. Preparation of Plant Extracts and Phytochemical Profiling

Plant extractions and phytochemical profiling of the plant extracts were obtained based on the standardized aqueous extraction method [22,28]. In this study, both varieties of MP *var. pumila* (MPvp) and *var. alata* (MPva) were collected from the Bujang Melaka Forest Reserve in Malaysia. A botanist, Mr. Sani Miran from the Faculty of Science, Universiti Kebangsaan Malaysia, authenticated the plant. The voucher specimens of MPva (voucher number: UKMB 30006/SM 2622) and MPvp (UKMB 30007/SM s.n) were deposited at the Herbarium of Universiti Kebangsaan Malaysia. The plants were separated into two varieties and each variety was further divided into two parts: leaves and roots (consisting of stems and roots). Each part was thoroughly washed and air-dried under shade. The dried materials were ground and weighed before use. The ground materials were extracted in distilled water at a 1:13 ratio of material to solvent for leaves and a 1:10 ratio for roots at 60 °C for 2 h using the reflux method. Each mixture was cooled and filtered. Then, the filtrate was freeze-dried overnight to sublimate the water in the frozen extract and to obtain the dried extract. The dried crude extracts were stored at −20 °C until further use.

Phytochemical profiling was performed on the crude extracts using liquid chromatography–tandem mass spectrometry (LC-MS/MS) based on the method described by Darmani, M. et al. [28]. Contents of selected compounds in the crude extracts were quantified from the calibration curves of six standard marker compounds, namely gallic acid, ellagic acid, caffeic acid, myricetin, apigenin, and quercetin, using LC-MS/MS. All standard calibration curves achieved good regression at >0.99.

### 2.2. Topical Ointment Preparation

Extracts were made into fine powder using a mortar and pestle. Fine particles have faster absorption rates and achieve better uniformity for ointment preparation. Cetomacrogol emulsifying ointment (Hovid Berhad, Malaysia) is a kind of paraffin used as a vehicle in these topical preparations. This paraffin is chemically inactive to the skin and is also inert and, therefore, would not react with the extracts. To optimize the concentration of plant extracts, a pilot study was conducted in which six different concentrations of 0.1%, 0.5%, 1.0%, 2.0%, 3.0%, and 4.0% from each extract were applied. The dose of 1.0% concentration for both leaf and root extracts of MPvp and 2.0% concentration for both leaf and root extracts of MPva exhibited the best effects to expedite for open-wound healing compared to the control group and other concentrations in the rat model. Based on the pilot study, 1.0% of MPvp leaf and root extracts and 2.0% of MPva leaf and root extracts were mixed with the vehicle to prepare the topical ointments [29]. Briefly, weighted paraffin and extract powder were put together into a clean glass plate and mixed with a spatula. Each mixing process of ointment was conducted three times to ensure uniformity. Then, the ointment was collected into a jar with a label and covered properly.

### 2.3. Experimental Animals

The Universiti Kebangsaan Malaysia Animal Ethics Committee approved all protocols of this animal experiment (project approval number: FP/FAR/2014/ISA/26-NOV./637-JAN.-2015-DEC.-2016). One hundred and twenty-six healthy male Sprague-Dawley rats were supplied by the Animal Research Center, Universiti Kebangsaan Malaysia, Titiwangsa, Kuala Lampur, Malaysia. All rats weighed between 200 and 250 g and were aged between 3 and 5 months. The rats were housed in plastic cages and acclimatized for one week to the laboratory environments with 22 ± 5 °C temperature, 80 ± 10% humidity, and 12-h day/night cycles. They were fed with food pellets (Gold Coin, Malaysia) and water, provided ad libitum. Rats were anesthetized intraperitoneally (IP) by injecting the cocktail preparation of ketamine hydrochloride (100 mg/mL) and xylazine hydrochloride (20 mg/mL) in a 1:1 ratio prior to all surgical procedures [30].

### 2.4. Excision Wound Model

The excision wound model, as described by Latif, M.A. et al. [31], was used in this study to determine the wound-healing effect. Before administering the anesthetic, the rats were weighed in advance. The weight readings were used to determine the volume of anesthetic injection (volume of injection). In this study, the volume of injection administered to each rat was 0.1 mL/100 g body weight. The injection was administered intraperitoneally (IP). Once anesthetized, hairs on the dorsal surface of rats were removed by an electric trimmer and softened, and the skin was disinfected using 70% alcohol and povidone–iodine solutions. Four wounds were made on the dorsal surface. Wounds were at a distance of 1.0 to 1.5 cm from each other. Each wound was punched to be 6 mm in diameter and 2 mm in thickness of skin. Wounds were punched on the dorsal surface to prevent scratching and biting by the rat itself. Treatment was started on the same day. The 126 male rats (Sprague-Dawley rats, 200–250 g body weight) were randomly and equally divided into 7 groups, namely sham-operated (SH), vehicle dressing (VD), flavine dressing (FD), MPvp leaves (PL), MPvp roots (PR), MPva leaves (AL), and MPva roots (AR) groups. The SH group was treated as the normal healing process, while the VD and GD groups were treated with emulsifying ointment and flavine, respectively, as controls. A 1.0% aqueous extract of MPvp leaves and roots was applied on the wounds of PL- and PR- treated groups and 2.0% aqueous extract of MPva leaves and roots was applied on the wounds of AL- and AR-treated groups. Wounds were dressed daily and 0.1 to 0.01 g of extracts was applied around on each wound once a day until complete healing. The percentage of wound contraction was measured on days 0, 2, 5, 8, and 9 and daily thereafter until complete wound healing. Photographs of the wounds were taken for macroscopic observation after each measurement of wound contraction. Six rats were taken from each group on days 2 and 8 for the histomorphological analysis using hematoxylin and eosin (H&E) staining, Masson–Goldner trichrome (MGT) staining, and immunohistochemistry (IHC) staining; antioxidant enzymes’ activities were assessed by measuring superoxide dismutase (SOD) and glutathione peroxidase (GPx) levels, and lipid peroxidation was assessed by determining malondialdehyde (MDA) levels.

### 2.5. Wound Healing Measurement

Macroscopic appearance and wound contraction were the two factors examined for excision wound healing observation. To examine macroscopic view and wound contraction, the wound area was calculated and photographs of the wound area on the skin were taken on day zero and the following 2, 5, 8, 9, 10, 11, 12, and 13 days after injury. A digital camera (Sony Cybershot, Japan) was used for taking photographs and digital caliper (General, China) was used for calculating wound size referred by the clock method [32]. The changes in wound size were calculated to determine the day the wound healed based on the equation by Ahmad, S.U. et al. [24].

### 2.6. Histological Analysis

On day 2 and day 8 after surgery, a full-thickness skin biopsy was taken from the center of wounds with surrounding tissues. Tissue samples were fixed in 10% neutral buffer formalin for histological analysis. After processing of tissue samples using a series of graded alcohol, these were embedded in paraffin wax. The tissues were sectioned at 5 µm for hematoxylin and eosin (H&E) and Masson–Goldner trichrome (MGT) staining. H&E staining was used to determine the skin microstructure including epithelialization, fibroblast proliferation, inflammation cell infiltration, vascularization, and granulation tissue formation [33]. MGT staining was performed according to the MGT kit protocol (Merck, Germany) to measure collagen density. Photomicrographs were analyzed using a microscopic image analyzer (Leica Microsystems, Germany). Stained sections were scored by histology experts in a blind fashion using the modified 0 to 3 numerical scale [34].

### 2.7. Immunohistochemistry Analysis

Immunohistochemistry (IHC) staining was performed using the mouse-specific Dako ARK™ (Animal Research Kit), the Peroxidase IHC staining kit (K3954, Agilent, Agilent, CA, USCalifornia, USA) for mouse monoclonal antibodies to fibronectin (ab6328, Abcam, Massachusetts, USA); collagen-III (ab6310, Abcam, Massachusetts, USA) and the rabbit-specific HRP/DAB detection IHC staining kit (ab80437, Abcam, UK) for rabbit polyclonal antibodies to fibroblast (ab28244, Abcam, USA), and collagen-I (ab34710, Abcam, Massachusetts, USA), according to the vendor’s protocol. The tissues for IHC were sectioned to 3-µm thickness. After being dewaxed in xylene and dehydrated in series of alcohol concentrations, tissues were treated with antigen retrieval solution (Agilent Dako, California, USA) using a microwave oven at maximum temperature to reduce non-specific antibody binding. Endogenous peroxidase activity and unwanted proteins were quenched using the peroxidase and protein blocks. Tissue slides were then incubated with different types of primary antibodies including anti-fibronectin and anti-fibroblast followed by incubation with HRP conjugate as a secondary antibody. Finally, incubation was done with the DAB substrate chromogen and counterstained with hematoxylin. Images were taken of IHC staining using a microscopic image analyzer (Leica Microsystems, Germany) at 20× magnification, which assessed the intensity of specific protein activity ranging from 0 (negative cells/tissues) to 3 (deeply stained cells/tissues) by the blind method [34].

### 2.8. Biochemical Analysis

Wound tissue samples were excised at the size of 1.0 cm × 1.0 cm on day 2 and day 8 post-wounding and biochemically analyzed to estimate endogenous antioxidant enzyme and lipid peroxidation activities. Each tissue sample was dried and weighed prior to use for analysis. Tissues were transferred to a bead tube with Tris-buffer and homogenized using a microtube homogenizer (Benchmark Scientific, USA). The homogenized tissues were then centrifuged at 6000 rpm for 20 min and we collected the supernatant for the biochemical estimations. Activity levels of endogenous antioxidant enzymes such as superoxide dismutase (SOD) (Item No 706002; Cayman) and glutathione peroxidase (GPx) (Item No 703102; Cayman) and lipid peroxidation, measured as malondialdehyde (MDA) content (Item No 10009055; Cayman), were measured following the vendor’s protocol.

### 2.9. Statistical Analysis

All quantitative data are presented as mean ± standard error of the mean (SEM) and were analyzed using Statistical Package for the Social Science (SPSS, version 23.0). Data obtained were evaluated by one-way ANOVA followed by Tukey’s HSD post-hoc test for statistical differences. *p* values < 0.05 were considered significant.

## 3. Results

### 3.1. Phytochemical Analysis

**Figure 1:**
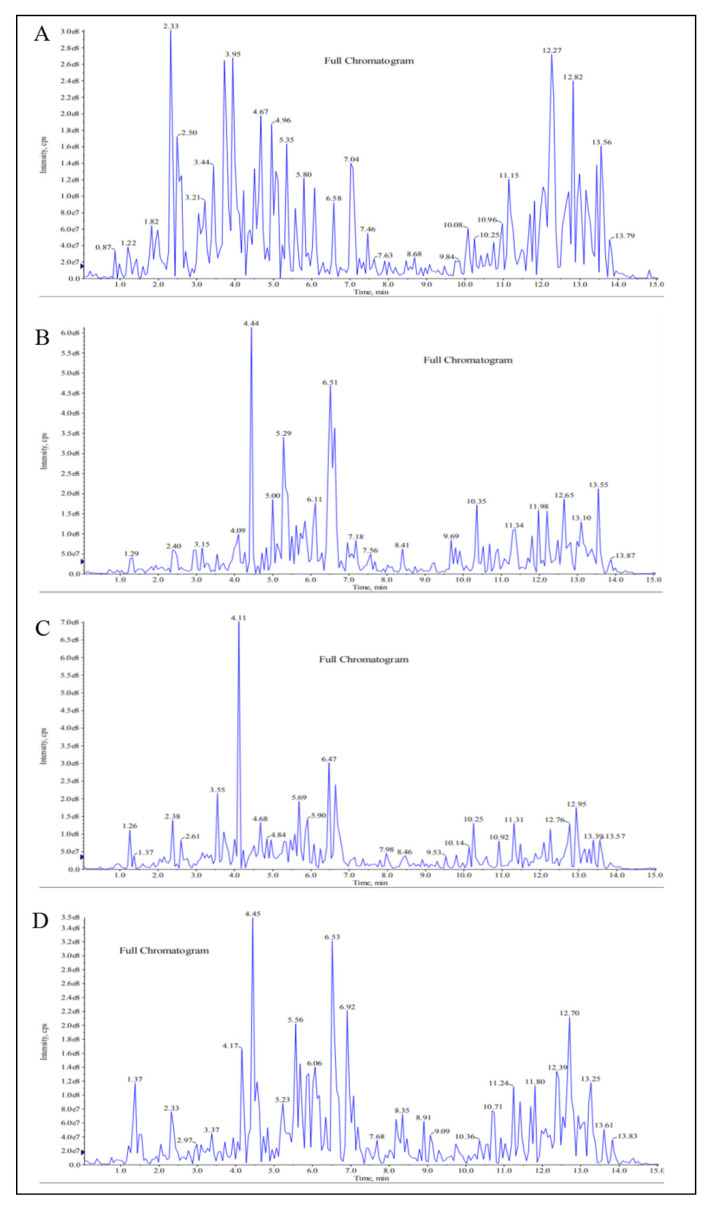
Liquid chromatography–tandem mass spectrometry (LC-MS/MS) chemical profiles of *Marantodes pumilum* (MP) aqueous extracts: (**A**) MP *var. pumila* (MPvp) leaves, (**B**) MPvp roots, (**C**) MP *var. alata* (MPva) leaves, and (**D**) MPva roots.

LC-MS/MS chromatographic profiles of the extracts are shown in Figure 1. Chromatographic profiling is a useful tool that provided preliminary comparative information on the chemical composition and complexity of the four extracts of different plant varieties and plant parts. Based on the MS database, several different compounds were characterized from the extracts, indicating that they have varied chemical compositions (Table 1). Only gallic acid and ellagic acid were determinable in all extracts, with MPvp leaf extract having the highest amount of gallic acid (6.81 mg/g) and MPvp root extract having the highest amount of ellagic acid (0.335 mg/g) (Table 2). However, only MPvp root extract had a quantifiable amount of caffeic acid (0.00052 mg/g). The contents of the other flavonoids were too low and not quantifiable using the LC-MS/MS method.

### 3.2. Determination of Wound Contraction

**Figure 2:**
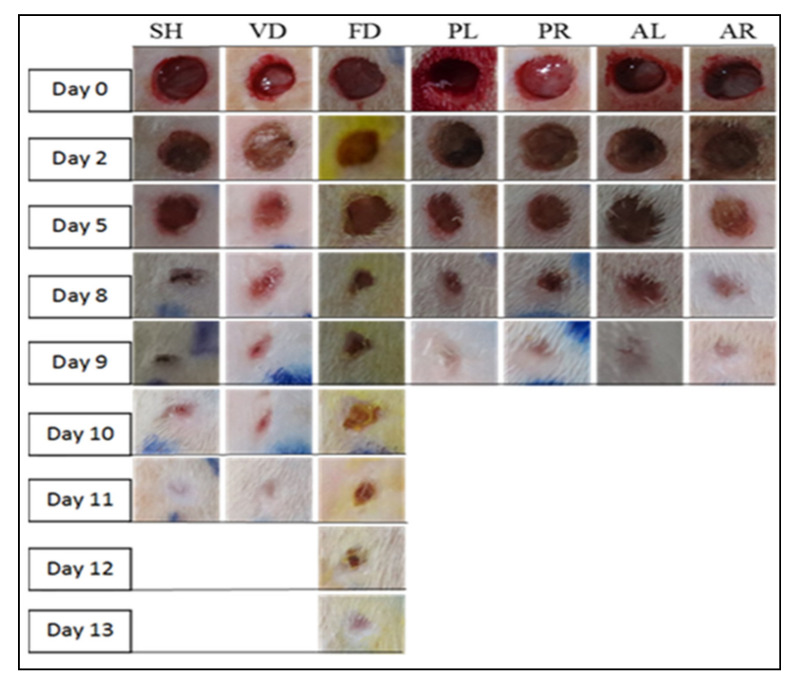
Macroscopic view of wounds of seven normal male rat experimental groups: SH, VD, FD, PL, PR, AL and AR on day 0, 2, 5, 8, 9, 10, 11, 12 and 13. SH: sham control group (without treatment), VD: treated with vehicle (Cetomacrogol ointment), FD: treated with flavine (Acriflavine 0.1%), PL: treated with 1.0% concentration of MPvp leaves extract, PR: treated with 1.0% concentration of MPvp stem-roots extract, AL: treated with 2.0% concentration of MPva leaves extract, AR: treated with 2.0% concentration of MPva stem-roots extract.

**Figure 3:**
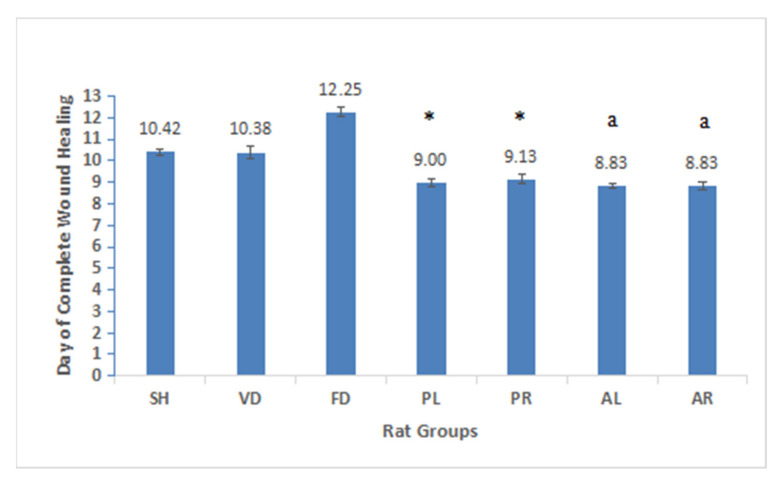
The day of complete wound healing seven male rat experimental groups: sham-operated SH, vehicle dressing (VD), flavine dressing (FD), MPvp leaves (PL), MPvp roots (PR), MPva leaves (AL), and MPva roots (AR). All data are given as mean ± S.E. for six animals in each group. Statistically significant results are indicated as (*) *p* < 0.05 versus untreated and control groups (SH, VD, and FD) and (a) *p* < 0.001 versus untreated and control groups (SH, VD, and FD). SH: sham control group (without treatment); VD: treated with vehicle (Cetomacrogol ointment); FD: treated with flavine (Acriflavine 0.1%); PL: treated with 1.0% concentration of MPvp leaves extract; PR: treated with 1.0% concentration of MPvp stem roots extract; AL: treated with 2.0% concentration of MPva leaves extract; AR: treated with 2.0% concentration of MPva stem roots extract.

The results of wound healing parameters, including macroscopic observation and wound healing measurement, in the study are presented in Figure 2 and Figure 3 and Table 3. In the macroscopic observations, it was noted that wound changes and healing patterns of the treatment groups were more advanced than the sham and control groups over the whole treatment period (Figure 2). Wounds treated with four different extracts (PL, PR, AL, and AR) healed on days 8 to 9, whereas the wounds of positive controls treated with flavine (FD) healed around day 13. A similar pattern of the wound healing process was shown between the sham and the negative control groups and both wounds healed approximately on day 11. Fluid secretion around the wound area was exhibited on days 1 to 2 for all the groups. However, a scab started to form in the treated groups on day 3 but was delayed in the sham and control groups. The scab started to remove from wound area on day 5 for the treated groups and was replaced by whitish fibrous tissue by days 8 to 9. An opposite trend was seen in the control groups in order to proliferate granulation tissues. It was proliferated between days 7 and 10 and followed by fibrous tissue growth on days 11 to 13. Figure 3 shows the mean value of the day of completed wound healing for all experimental groups. The healing rate of groups treated with PL and PR (*p* < 0.05) and AL and AR extracts (*p* < 0.001) was significantly higher than that of the untreated and control groups (SH, VD, and FD). Among the four treated groups (PL, PR, AL, and AR), there were no significant differences in terms of the day of complete wound healing, which was also similar among the untreated and control groups.

### 3.3. Histological Evaluation

**Figure 4:**
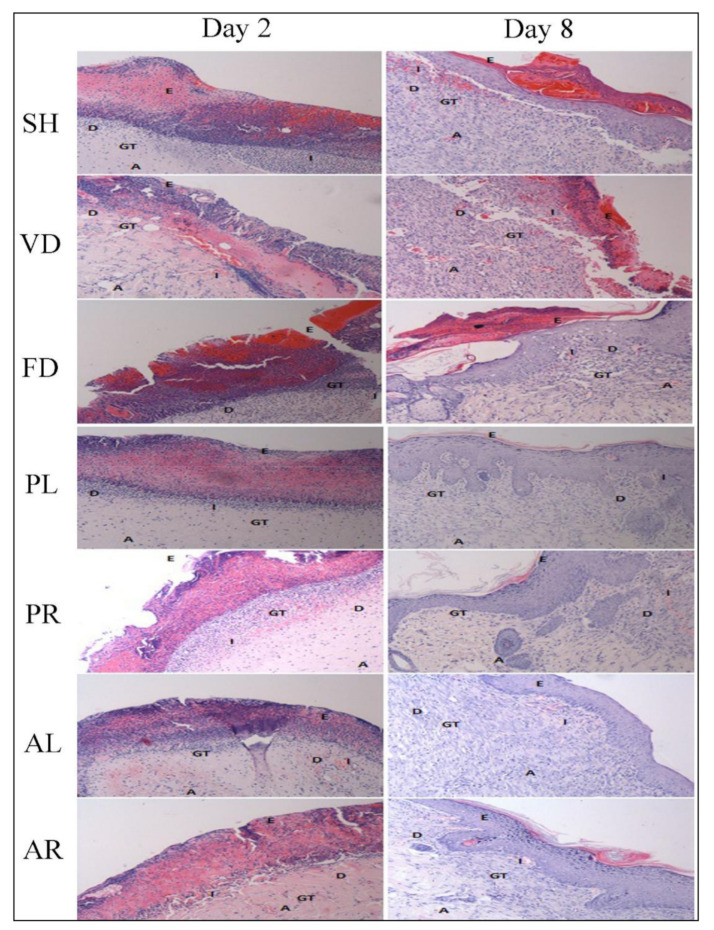
Histopathological view of skin wounds by hematoxylin and eosin (H&E) staining of seven male rat experimental groups: SH, VD, FD, PL, PR, AL, and AR. Pictures of stain are at 10x magnification. SH: sham control group (without treatment); VD: treated with vehicle (Cetomacrogol ointment); FD: treated with flavine (Acriflavine 0.1%); PL: treated with 1.0% concentration of MPvp leaves extract; PR: treated with 1.0% concentration of MPvp stem roots extract; AL: treated with 2.0% concentration of MPva leaves extract; AR: treated with 2.0% concentration of MPva stem roots extract. E: Epidermis; D: Dermis; F: Fibroblast cells; I: Inflammatory cells; A: Angiogenesis; G: Granulation tissue. Bar scale: 20 µm.

**Figure 5:**
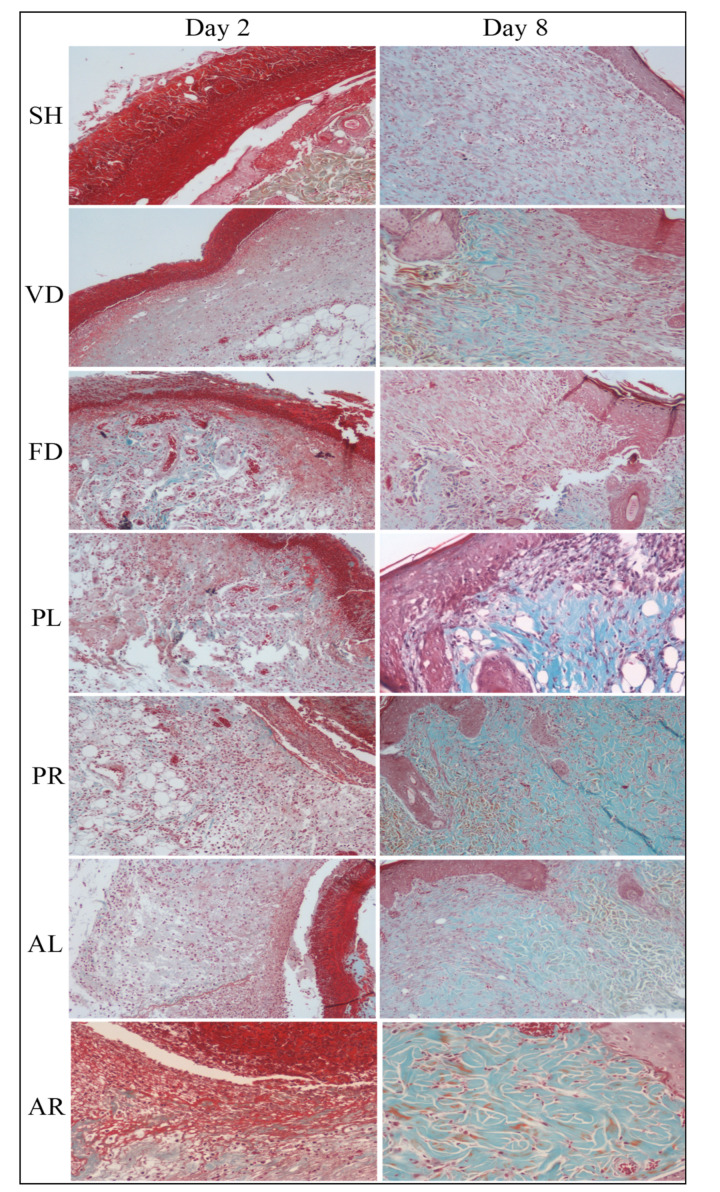
Histopathological view of collagen deposition in wounded skin tissue evaluated by Masson–Goldner trichrome (MGT) staining of seven male rat experimental groups: SH, VD, FD, PL, PR, AL, and AR. Pictures of stain are at 20× magnification. Blue staining indicates collagen deposition. SH: sham control group (without treatment); VD: treated with vehicle (Cetomacrogol ointment); FD: treated with flavine (Acriflavine 0.1%); PL: treated with 1.0% concentration of MPvp leaves extract; PR: treated with 1.0% concentration of MPvp stem roots extract; AL: treated with 2.0% concentration of MPva leaves extract; AR: treated with 2.0% concentration of MPva stem roots extract. Bar scale: 20 µm.

Histopathological examination of the wound tissues was carried out using hematoxylin and eosin (H&E) for evaluation of skin morphology and Masson–Goldner trichrome (MGT) staining for estimation of collagen deposition. In Table 4, the histological findings are similar for each group in terms of regeneration of epithelial cells, granulation tissue formation, proliferation of fibroblast cells, and neovascularization, except for inflammatory cell infiltration which demonstrated a different trend. At the early stage (day 2), parameters of histology such as proliferation of epithelial cells, vascular cells, and granulation tissues in wounded skin were immature and the score was around 1. By day 8, most of the parameters were matured in the treated groups with a score of 2 to 3, whereas the control groups were less matured than treated groups on the corresponding days. An opposite trend has been seen in the inflammatory cell infiltration. The score of inflammatory cells was higher at the early stage and lower at the later stage (day 8) in both the control and the treated groups on corresponding days. The scores of three treated groups (PR, AL, and AR) were significantly different compared to the FD group. Figure 4 shows the histomorphological view of the wounded skin by H&E staining of the SH, VD, FD, PL, PR, AL, and AR groups on days 2 and 8 post-wounding. The pictures of skin histology illustrated that on day 2, there were many inflammatory cells on necrotic cells layer. The skin microstructure at the wound site such as epidermal, dermal, and hypodermal was not prominent at an early stage, as indicated by the lacking proliferation of epithelial cell, fibroblast, and granulation tissue formation. However, on day 8, inflammatory cells still appeared in the wounding area and the epidermal layer was not mature enough in the control groups whereas these were improved significantly in the treated groups. The wound healing process in the treated groups was recovered by epithelialization with the formation of keratinocytes at the same time. Newly formed capillary, fibroblast, collagen, and connective tissues were seen in the treated groups on day 8, whereas the formation of fibroblast cells, granulation tissue, and, eventually, the re-epithelialization of wound skin in the control groups were delayed and formed on days 11 to 13. Overall, the skin structure was more matured in the treated groups compared to the control groups at the later stage. Collagen deposition by MGT staining of the experimental groups was scant at the early stage (Table 3). However, on day 8, the score was significantly higher in the treated groups compared to the untreated and control groups (*p* < 0.05 versus SH, VD, and FD). The score for the treated groups was nearly 2.58 ± 0.20, while that of the FD control group was approximately 1.42 ± 0.20. Figure 5 demonstrates that collagen deposition in the wounded skin tissue evaluated by MGT staining on days 2 and 8 post-wounding. At the early stage (day 2), the appearance of collagen was poor for all the groups. Collagen deposition enhanced with time. It was mature and significantly increased in all the treated groups on day 8, whereas it was mature but less significant in the control groups.

### 3.4. Immunohistochemistry (IHC) Analysis

**Figure 6:**
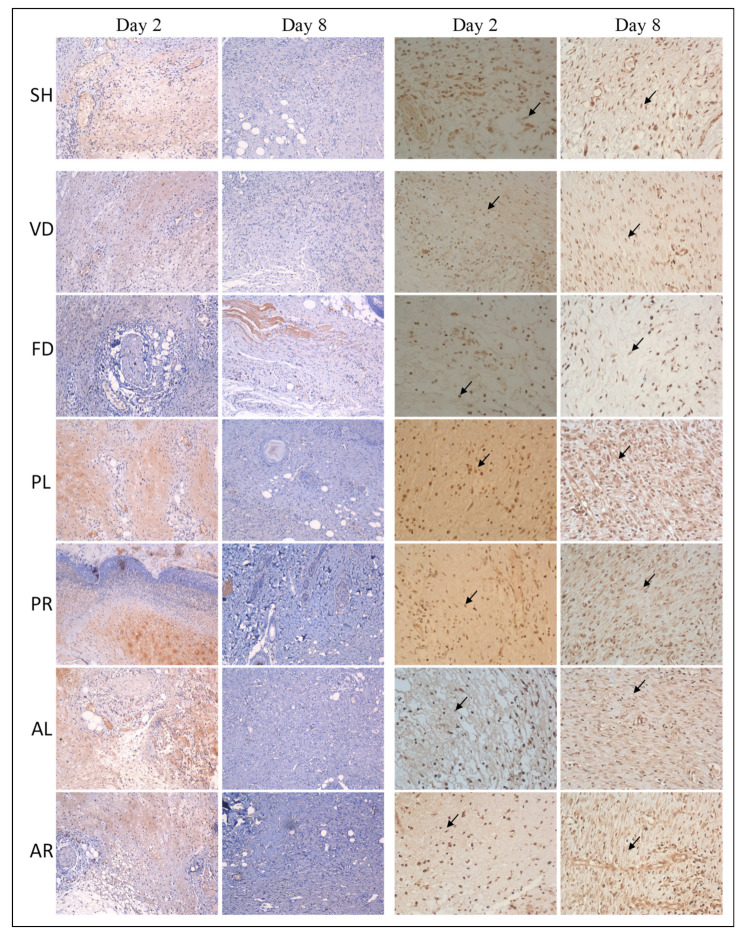
Photomicrographs of wounded skin tissue immunostained with an antibody against fibronectin (10× magnification) and fibroblast (20× magnification) in seven male rat experimental groups: SH, VD, FD, PL, PR, AL, and AR. Brown staining in the first and second columns indicates fibronectin contents, and arrows in the third and fourth columns indicate fibroblast cells. SH: sham control group (without treatment); VD: treated with vehicle (Cetomacrogol ointment); FD: treated with flavine (Acriflavine 0.1%); PL: treated with 1.0% concentration of MPvp leaves extract; PR: treated with 1.0% concentration of MPvp stem roots extract; AL: treated with 2.0% concentration of MPva leaves extract; AR: treated with 2.0% concentration of MPva stem roots extract. Bar scale: 50 µm.

**Figure 7:**
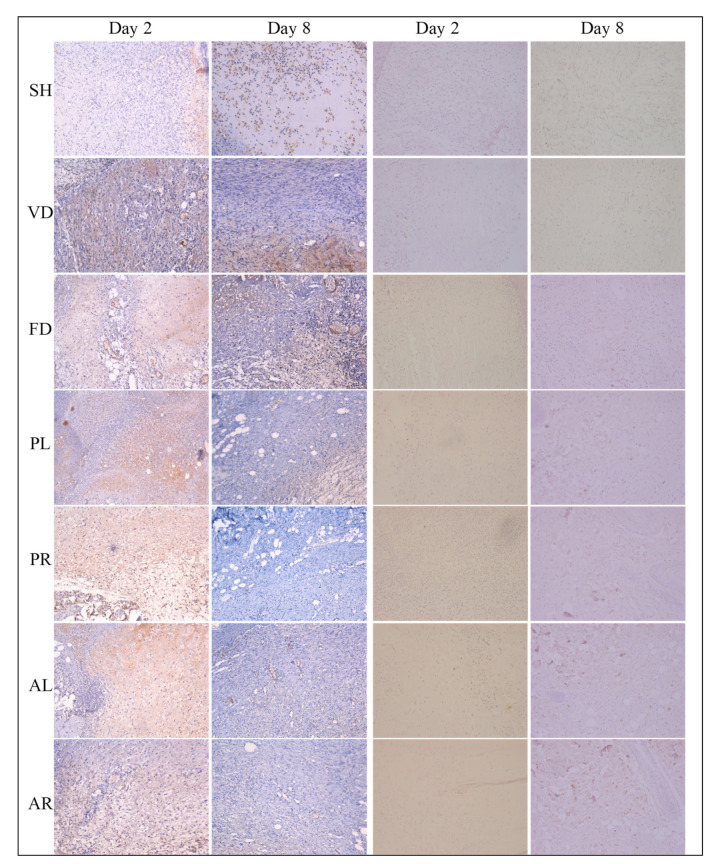
Photomicrographs of wounded skin tissue immunostained with an antibody against collagen-III (10× magnification) and collagen-I (10× magnification) in seven male rat experimental groups: SH, VD, FD, PL, PR, AL, and AR. Brown staining in the first and second columns indicates collagen-III deposition and the third and fourth columns indicate collagen-I transformation. SH: sham control group (without treatment); VD: treated with vehicle (Cetomacrogol ointment); FD: treated with flavine (Acriflavine 0.1%); PL: treated with 1.0% concentration of MPvp leaves extract; PR: treated with 1.0% concentration of MPvp stem roots extract; AL: treated with 2.0% concentration of MPva leaves extract; AR: treated with 2.0% concentration of MPva stem roots extract. Bar scale: 50 µm.

Table 5 showed the scores for fibronectin, fibroblast, collagen-III, and collagen-I antibodies obtained by IHC staining of all the groups at day 2 and day 8 after wound induction. The scores indicate the intensity of antibodies’ expression in the wounded skin tissue. The score of fibronectin was from 2.58 ± 0.20 to 3.00 ± 0.0 for the treated groups on day 2, whereas the reactivity decreased with extended time, and the score was less than one for all the groups on day 8. The intensity of fibronectin and collagen-III expressions was significantly higher at the later stage (day 8) in treated groups (*p* < 0.05) compared to the untreated and control groups. Figure 6 indicates that the intensity of fibronectin was extensive in almost all of the groups. It decreased with extended time. Collagen-III was less abundant in the control groups on day 8, whereas there was minimal appearance in the treated groups (Figure 7). In contrast, an opposite trend was seen in both the score and intensity of fibroblast and collagen-I. At the early stage (day 2), the intensity of fibroblast and collagen-I expressions was lower for all the groups (Figure 6 and Figure 7). However, the reactivity was enhanced with extended time and varied between each group. However, there was no significant difference in the scores of fibroblast and collagen-I intensity on day 8 between the control and treated groups.

### 3.5. Superoxide Dismutase (SOD) Enzyme Activity

**Figure 8:**
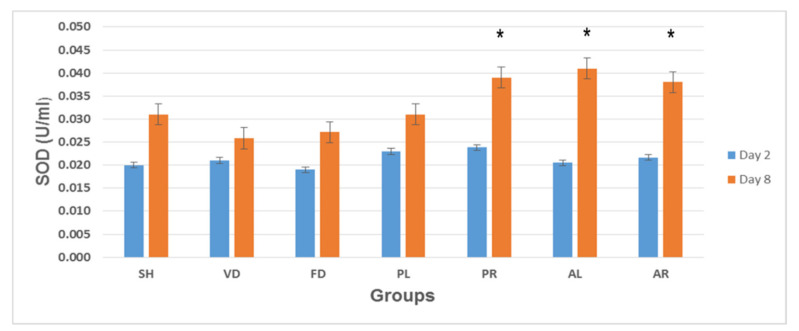
Superoxide dismutase (SOD) levels in skin tissue of seven rat experimental groups: SH, VD, FD, PL, PR, AL, and AR. All data are given as mean ± S.E. for six animals in each group. Statistically significant results indicated as (*) *p* < 0.05 versus control groups (SH, VD, and FD) on the corresponding day. SH: sham control group (without treatment); VD: treated with vehicle (Cetomacrogol ointment); FD: treated with flavine (Acriflavine 0.1%); PL: treated with 1.0% concentration of MPvp leaves extract; PR: treated with 1.0% concentration of MPvp stem roots extract; AL: treated with 2.0% concentration of MPva leaves extract; AR: treated with 2.0% concentration of MPva stem roots extract.

Figure 8 shows the mean values of SOD levels in skin tissue on post-wounded day 2 and day 8 of the seven rat experimental groups: SH, VD, FD, PL, PR, AL, and AR. At the early stage of wound treatment (day 2), the SOD levels ranged from 0.019 ± 0.003 to 0.024 ± 0.004 U/mL, and there were no significant differences between the groups. The SOD levels increased gradually after day 2 for all the groups. On day 8, the SOD levels in three treated groups (PR = 0.039 ± 0.002 U/mL; AL = 0.041 ± 0.002 U/mL; and AR = 0.038 ± 0.002 U/mL) were significantly higher than the SH (0.031 ± 0.002 U/mL), VD (0.026 ± 0.002 U/mL), FD (0.027 ± 0.002 U/mL), and PL (0.031 ± 0.002 U/mL) groups.

### 3.6. Glutathione Peroxidase (GPx) Enzyme Activity

**Figure 9:**
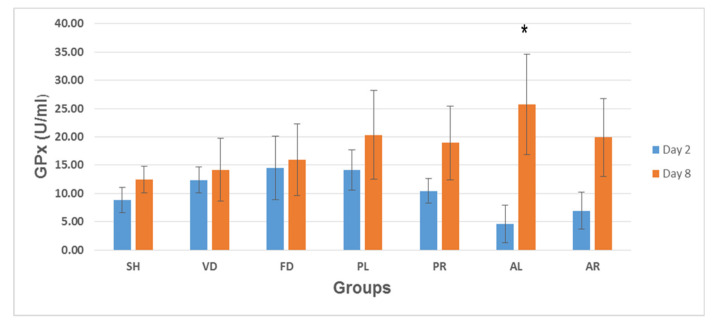
Glutathione peroxidase (GPx) levels in skin tissue of seven rat experimental groups: SH, VD, FD, PL, PR, AL, and AR. All data are given as mean ± S.E. for six animals in each group. Statistically significant results indicated as (*) *p* < 0.05 versus control groups (SH, VD, and FD) on the corresponding day. SH: sham control group (without treatment); VD: treated with vehicle (Cetomacrogol ointment); FD: treated with flavine (Acriflavine 0.1%); PL: treated with 1.0% concentration of MPvp leaves extract; PR: treated with 1.0% concentration of MPvp stem-roots extract; AL: treated with 2.0% concentration of MPva leaves extract; AR: treated with 2.0% concentration of MPva stem-roots extract.

Figure 9 shows the mean values of GPx levels in skin tissue on post-wounded day 2 and day 8 of the seven rat experimental groups: SH, VD, FD, PL, PR, AL, and AR. Similar to the trend of SOD levels during the early stage of wound treatment (day 2), GPx levels ranged from 4.58 ± 3.30 to 14.50 ± 5.63 U/mL and there were no significant differences among the groups. After day 2, GPx levels increased gradually for all the groups. At day 8, GPx levels in the AL-treated group (AL = 25.78 ± 8.89 U/mL) were significantly higher than in the SH (12.45 ± 2.34 U/mL), VD (14.18 ± 5.54 U/mL), and FD (15.96 ± 6.37 U/mL) groups. However, there were no significant differences between the other treated groups (PL = 20.35 ± 7.85 U/mL; PR = 18.92 ± 6.57 U/mL; and AR = 19.89 ± 6.89 U/mL) and control groups or between the treated groups.

### 3.7. Lipid Peroxidation Analysis: Malondialdehyde (MDA) Measurement

**Figure 10:**
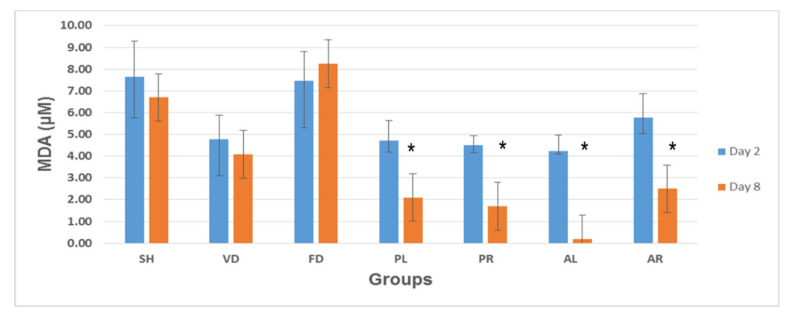
Malondialdehyde (MDA) levels in skin tissue of seven rat experimental groups: SH, VD, FD, PL, PR, AL, and AR. All data are given as mean ± S.E. for six animals in each group. Statistically significant results indicated as (*) *p* < 0.05 versus control groups (SH, VD, and FD) on the corresponding day. SH: sham control group (without treatment); VD: treated with vehicle (Cetomacrogol ointment); FD: treated with flavine (Acriflavine 0.1%); PL: treated with 1.0% concentration of MPvp leaves extract; PR: treated with 1.0% concentration of MPvp stem-roots extract; AL: treated with 2.0% concentration of MPva leaves extract; AR: treated with 2.0% concentration of MPva stem-roots extract.

Figure 10 shows the mean values of MDA levels in skin tissue on post-wounded day 2 and day 8 of the seven rat experimental groups: SH, VD, FD, PL, PR, AL, and AR. At the early stage of wound treatment (day 2), MDA levels showed an opposite trend to that of SOD and GPx levels in wounded skin tissue among the groups. At day 2, the MDA levels were higher than on day 8. The VD (4.78 ± 1.09 μM), PL (4.70 ± 0.92 μM), PR (4.50 ± 0.44 μM), and AL (4.23 ± 0.74 μM) groups had almost similar MDA levels on day 2. The SH (7.63 ± 1.65 μM), FD (7.47 ± 1.35 μM), and AR (5.77 ± 1.12 μM) groups contained an almost equal amount of MDA on the same day. At the late stage (day 8), the MDA levels were decreased for all the groups except for the FD group. In the FD group, MDA levels were increased from 7.47 ± 1.35 μM on day 2 to 8.25 ± 2.17 μM on day 8. On day 8, MDA levels were significantly reduced in all treated groups compared to all control groups (*p* < 0.05).

## 4. Discussion

Cutaneous wound healing is a major interest for public health because it is the most common morbidity in the daily life of people. Natural medicinal plants have become a reliable source for therapeutic agents. However, scientific evidence on topical agents and their therapeutic effects on skin wound healing is limited [35]. Groups using the aqueous extracts of MP showed faster healing and earlier wound contraction compared with untreated and control groups, relating to MP’s various biological effects on different phases of the wound healing process. Visual observation indicated that wounds treated with MP form scabs earlier than those in the untreated and control groups, consuming the fluid secretion rapidly from the inflammatory cells. The results suggested that MP has anti-inflammatory activities which can resolve the inflammatory process in wounds to accelerate the healing process. Persistence of inflammation delays the healing process and induces a pathological condition [36]. The formation of scabs in the MP-treated groups started from day 3 and they were covered with new epithelial cells shortly after. Scabs in the untreated and control groups were retained for longer time, which prevented proliferation of new epithelial cells, eventually delaying the healing process [37]. The proliferation phase starts with the formation of scab in skin tissue, where many cellular events are involved in the contraction of wound area [6]. Although wounds observed on day 9 and day 13 were found to be healed macroscopically in the study, the proliferation phase of wound healing could take up to three months and the modeling phase will continue for several months after the wound is formed [38].

The histopathological findings of re-epithelization activity, inflammatory cell infiltration, fibroblast cell proliferation, angiogenesis, fibronectin fiber formation, collagen deposition, and granulation tissue formation are good indicators for the wound contraction process by MP. In the current study, inflammatory cells were significantly higher at the early stage and diminished at the later stage of wound healing. The results correlate with a previous study that postulated inflammatory cell infiltration at the injury site after forming a stable clot in the dermal tissues [39]. Neutrophils are the first inflammatory cells that arrive at the wound site to eliminate microorganisms and initiate the inflammatory process [40]. Neutrophils prepare the wound bed for healing by removing necrotic tissue, debris, and bacterial contaminants as well as deriving growth factors and activating fibroblasts. The process of angiogenesis occurs concurrently with fibroblast proliferation when endothelial cells migrate to the wound area [41]. As fibroblast and epithelial cell activities require oxygen and nutrients, angiogenesis is imperative for other stages of wound healing such as epidermal and fibroblast migration. Our H&E findings showed that even though fibroblast and vascular endothelial cells appeared from the early stage, their numbers increased significantly at the later stage of wound healing in the treated groups compared to the untreated and control groups. Our histological analysis of fibroblast intensity also showed a similar trend. A study suggested that fibroblasts begin entering the wound site during the late inflammatory phase [42]. Usually, fibroblasts are derived from the adjacent uninjured cutaneous tissue to the wound site. They can also be derived from blood-borne circulating adult stem cells/precursors [43]. At the late stage of the inflammatory phase, fibroblasts migrate to the wound site adhered to fibrin through fibronectin [44]. This study showed that the intensity of fibronectin in the treated groups was higher than in the control groups at the early stage, whereas the intensity decreased significantly in the treated groups at the later stage of wound healing. These results are supported by a previous study which demonstrated that fibronectin has profound effects on the wound healing process [45]. It can induce the growth and migration of extracellular matrix during the development and organization of granulation tissue [46]. During the early stage of wound healing, fibroblasts are deposited into the wound bed and produce collagen for migration toward the wound site [47]. Collagen deposition is important because it increases the strength of the wound. Most of the collagen found in skin is type-I and type-III. Collagen-III appeared at the beginning of the wound healing process and was replaced by the stronger type-I collagen during the proliferation and maturation phases [48]. It controls many cellular functions, including cell shape and differentiation [49,50], migration [51], and synthesis of a number of proteins [52]. The current study, in both MGT and IHC analyses, also showed similar results. The findings in the study demonstrated that collagen became denser at the later stage of wound healing. Granulation tissue consists of many fibers such as collagen, extracellular matrix, blood vessels, and various cells such as inflammatory cells, fibroblasts, myofibroblasts, and endothelial cells. These components will be aligned and rearranged in the maturation and remodeling phase [53]. At the end of the granulation phase, fibroblasts begin to undergo apoptosis, converting granulation tissue from an environment rich in cells to one that consists mainly of collagen [2]. Epithelization induces proliferation and migration of epithelial cells across the wound bed [54]. Therefore, a higher re-epithelization score in the MP extracts groups might be due to facilitated proliferation of epithelial cells and/or increasing viability of epithelial cells [55].

Reactive oxygen species (ROS) or free radicals are also major contributors to oxidative stress, which will delay wound healing [56]. Leukocytes and many inflammatory cells, including neutrophils and macrophages, release ROS at wound sites [57,58]. Overproduction of ROS causes breakdown of collagen fiber and extracellular matrix, which leads to chronic wounds [9,59]. Many cellular enzymatic antioxidants such as SOD and GPx hasten the process of wound healing by destroying free radicals [60]. The SOD and GPx levels of the treated groups were significantly higher compared to the untreated and control groups at the late stage of wound healing. This suggests that the antioxidant activity of MP may enhance its wound healing activity. The excessive production of ROS also induces lipid peroxidation which affects various cellular functions such as granulation tissue formation, collagen and fibroblast metabolism, angiogenesis, and epithelialization [61]. The significant reduction in MDA levels, an index of lipid peroxidation, in the granulation tissue may accelerate the wound healing process. Several studies have shown that antioxidant enzymes can reduce free radicals in the body and maintain lipid peroxidation [62]. MP has antioxidative scavenger activities which are effective for reducing lipid peroxidation during wound healing. The antioxidative properties of MP may be contributed to by its phytochemical contents such as gallic acid, ellagic acid, and caffeic acid. Gallic acid has valuable antioxidant properties by revealing free-radical-neutralizing capabilities [63]. Ellagic acid has also been demonstrated to possess a strong ability to scavenge free radicals both in vivo and in vitro [64,65]. The antioxidant activity of phenolic agents is mostly because of the redox attributes that let them act as hydrogen donors, singlet oxygen quenchers, and reducing mediators [66]. Phytochemicals may enhance the wound healing process by neutralizing oxygen anions and inhibiting peroxyl-free radicals [67]. MP has a role in the wound healing process as indicated by the presence of its phytochemicals.

## 5. Conclusions

MP facilitated the wound healing process in the male rat model. A histological analysis among MP-treated groups revealed better re-epithelialization, enhanced fibronectin content and fibroblast cells, as well as higher fiber transformation from collagen-III to collagen-I accompanied with an abatement of inflammatory cells in the granulation tissues. Moreover, MP administration caused a significant increase in enzymatic antioxidant activities and a decline in lipid peroxidation. It can be proposed that the high content of phenolic compounds in the MP extracts may be responsible for their antioxidative properties. Its high antioxidant activity suggests that the plant can be used as an effective wound-healing agent. Further studies with purified constituents are needed to understand the complete mechanism of wound healing activity using MP extracts.

## Figures and Tables

**Table 1:** Detection of phytochemical compounds in MP aqueous extracts using LC-MS/MS.

Extracts	Retention Time (min)	[M − H]^−^ *m*/*z*, (Da)	[M + H]^+^ *m*/*z*, (Da)	Fragment Ions	Detected Chemical Constituents
MPvp leaves	1.43		165	147, 105, 75	Hydroxylated cinnamic acid
	1.76	331		303, 211, 192, 169, 124	Monogalloyl glucose isomer
	1.88	191		173, 129, 110, 87	Quinic acid
	2.13	169		124, 95, 68	Gallic acid
	2.72	483		331, 169, 124	Digalloyl glucose
	2.85	179		135, 107	Caffeic acid
	3.11	301		257, 229, 185	Ellagic acid
	3.72	470		452, 318, 300, 256, 169	Gallic acid derivative
	3.77	136		108	p-hydroxybenzoic acid
	3.99	451		433, 361, 289, 165	Catechin-7-O-glucoside
	4.28	147		118, 102	Cinnamic acid
	4.39	289		245, 201, 181	Catechin
	4.95	463		373, 316, 300, 271, 179	Myricetin derivative
	5.23	331		287, 271, 211, 179, 150	Monogalloyl glucose
	6.58		327	309, 201, 171	Methyl-2-[cyclohex-2-en-1-yl(hydroxy)methyl]-3-hydroxy-4-(2-hydroxyethyl)-3-methyl-5-oxoprolinate
MPvp roots	2.12	169		124, 95, 68	Gallic acid
	2.37	315		195, 163, 152, 123	Protocatechuic acid hexoside
	2.84	179		135, 107	Caffeic acid
	2.98	153		123, 108	Protocatechuic Acid
	3.12	301		257, 229, 185	Ellagic acid
	5.00	483		313, 169, 139	Digalloyl glucose
	6.18	329		311, 201, 171	3,30-di-O-methyl ellagic acid
MPva leaves	2.12	169		124, 95, 68	Gallic acid
	2.94	179		135, 107	Caffeic acid
	3.05	153		123, 108, 66	Protocatechuic acid
	3.13	301		257, 229, 185	Ellagic acid
	4.00		636	484, 465, 313, 169	Gallic acid derivative
	4.79	136		92, 64	p-hydroxybenzoic acid
	5.24	331		287, 271, 179, 151	Monogalloyl glucose
	5.69		327	309, 201, 171, 137	Methyl-2-[cyclohex-2-en-1-yl(hydroxy)methyl]-3-hydroxy-4-(2-hydroxyethyl)-3-methyl-5-oxoprolinate
	5.91	329		311, 211, 171, 139	3,30-di-O-methyl ellagic acid
	6.64		327	291, 211, 171	Methyl-2-[cyclohex-2-en-1-yl(hydroxy)methyl]-3-hydroxy-4-(2-hydroxyethyl)-3-methyl-5-oxoprolinate isomer
MPva roots	1.37	377		341, 215, 179	Caffeic acid derivative
	1.49	191		172, 111, 84	Quinic acid
	2.13	169		124, 95, 68	Gallic acid
	2.65	153		123, 108, 64	Protocatechuic acid
	2.84	179		135, 107	Caffeic acid
	3.11	301		257, 229, 185	Ellagic acid
	4.16	289		245, 225, 209, 137	Catechin

**Table 2:** Content of phenolic acids and polyphenol in MP aqueous extracts by using LC-MS/MS.

Extracts	Content (mg/g)		
	Gallic Acid	Caffeic Acid	Ellagic Acid
MPvp leaves	6.810	<0	0.0585
MPvp roots	4.080	0.00052	0.335
MPva leaves	5.540	<0	0.0701
MPva roots	2.240	<0	0.115

**Table 3:** Macroscopical observation of seven male rat experimental groups: SH, VD, FD, PL, PR, AL, and AR on a daily basis. SH: sham control group (without treatment); VD: treated with vehicle (Cetomacrogol ointment); FD: treated with flavine (Acriflavine 0.1%); PL: treated with 1.0% concentration of MPvp leaves extract; PR: treated with 1.0% concentration of MPvp stem roots extract; AL: treated with 2.0% concentration of MPva leaves extract; AR: treated with 2.0% concentration of MPva stem roots extract.

Macroscopic Observations	Day						
	SH	VD	FD	PL	PR	AL	AR
Excision	0	0	0	0	0	0	0
Secreting fluid	1–3	1–3	1–3	1–2	1–2	1–2	1–2
Scab	4–7	4–7	4–9	3–5	3–5	3–5	3–5
Granulation tissue	8–9	8–9	10–11	6–8	6–8	6–7	6–7
Fibrous	10–11	10–11	12–13	9	9	8–9	8–9

**Table 4:** Mean score from histological evaluation by hematoxylin and eosin (H&E) and estimation of collagen deposition by Masson–Goldner trichome (MGT) staining of seven male rat experimental groups: SH, VD, FD, PL, PR, AL, and AR. All data are given as mean ± S.E. for six animals in each group. Statistically significant results are indicated as (*) *p* < 0.05 versus untreated and control groups (SH, VD, and FD) and (a) *p* < 0.05 versus FD group. SH: sham control group (without treatment); VD: treated with vehicle (Cetomacrogol ointment); FD: treated with flavine (Acriflavine 0.1%); PL: treated with 1.0% concentration of MPvp leaves extract; PR: treated with 1.0% concentration of MPvp stem-roots extract; AL: treated with 2.0% concentration of MPva leaves extract; AR: treated with 2.0% concentration of MPva stem-roots extract.

Groups	Re-Epithelialization		Inflammatory Cell Infiltration		Fibroblast Cell Proliferation		Neo-Vascularization		Granulation Tissue Formation		Collagen Deposition	
	Day 2	Day 8	Day 2	Day 8	Day 2	Day 8	Day 2	Day 8	Day 2	Day 8	Day 2	Day 8
SH	0.33 ± 0.21	2.17 ± 0.11	2.00 ± 0.26	1.50 ± 0.22	1.77 ± 0.17	2.17 ± 0.17	0.50 ± 0.22	1.83 ± 0.31	1.67 ± 0.21	2.67 ± 0.21	1.00 ± 0.00	1.58 ± 0.15
VD	0.33 ± 0.21	2.17 ± 0.31	2.00 ± 0.26	1.67 ± 0.21	1.33 ± 0.21	2.33 ± 0.21	0.83 ± 0.17	2.00 ± 0.26	1.67 ± 0.21	2.17 ± 0.17	0.67 ± 0.21	1.67 ± 0.17
FD	0.33 ± 0.21	2.08 ± 0.15	2.00 ± 0.26	1.83 ± 0.17	1.67 ± 0.33	2.33 ± 0.21	0.67 ± 0.21	1.67 ± 0.21	1.67 ± 0.21	2.00 ± 0.00	0.67± 0.21	1.42 ± 0.20
PL	0.33 ± 0.21	2.83 ± 0.11 *	1.83 ± 0.17	1.00 ± 0.22	1.17 ± 0.17	2.67 ± 0.21	0.92 ± 0.08	2.75 ± 0.17 ^a^	2.00 ± 0.26	2.33 ± 0.21	0.83± 0.11	2.67 ± 0.17 *
PR	0.50 ± 0.22	2.92 ± 0.08 *	1.83 ± 0.31	0.92 ± 0.08 ^a^	1.33 ± 0.21	2.67 ± 0.21	1.17 ± 0.17	2.58 ± 0.20	2.33 ± 0.21	2.50 ± 0.22	1.00± 0.00	2.58 ± 0.20 *
AL	0.67 ± 0.33	3.00 ± 0.00 *	1.83 ± 0.17	0.67 ± 0.17 ^a^	1.17 ± 0.17	3.00 ± 0.00 *	1.17 ± 0.17	2.75 ± 0.17 ^a^	2.00 ± 0.00	2.83 ± 0.17 ^a^	1.17 ± 0.17	2.58 ± 0.20 *
AR	0.17 ± 0.17	2.92 ± 0.08 *	1.33 ± 0.21	0.67 ± 0.21 ^a^	1.83 ± 0.17	2.67 ± 0.21	0.92 ± 0.08	2.42 ± 0.20	2.00 ± 0.00	2.83 ± 0.17 ^a^	1.00 ± 0.00	2.58 ± 0.20 *

**Table 5:** Mean score of proteins expression obtained by immunohistochemistry (IHC) staining of seven male rat experimental groups: SH, VD, FD, PL, PR, AL, and AR. All data are given as mean ± S.E. for six animals in each group. Statistically significant results are indicated as (*) *p* < 0.05 versus untreated and control groups (SH, VD, and FD) on the same day. SH: sham control group (without treatment); VD: treated with vehicle (Cetomacrogol ointment); FD: treated with flavine (Acriflavine 0.1%); PL: treated with 1.0% concentration of MPvp leaves extract; PR: treated with 1.0% concentration of MPvp stem roots extract; AL: treated with 2.0% concentration of MPva leaves extract; AR: treated with 2.0% concentration of MPva stem roots extract.

Score of Proteins Expression	Day	SH	VD	FD	PL	PR	AL	AR
Fibronectin	Day 2	1.58 ± 0.15	1.17 ± 0.17	0.92 ± 0.17	2.92 ± 0.08	2.83 ± 0.11	3.00 ± 0.00	2.58 ± 0.20
	Day 8	1.20 ± 0.11	1.17 ± 0.21	1.63 ± 0.17	0.40 ± 0.15 *	0.50 ± 0.17 *	0.50 ± 0.17 *	0.83 ± 0.11 *
Fibroblast	Day 2	1.17 ± 0.11	1.33 ± 0.21	1.17 ± 0.11	2.17 ± 0.17	2.67 ± 0.21	2.50 ± 0.22	2.33 ± 0.21
	Day 8	2.17 ± 0.17	2.42 ± 0.20	2.00 ± 0.00	3.00 ± 0.00	2.92 ± 0.08	3.00 ± 0.00	2.83 ± 0.11
Collagen-III	Day 2	2.20 ± 0.15	2.10 ± 0.17	2.58 ± 0.20	2.67 ± 0.17	2.70 ± 0.20	2.72 ± 0.20	2.58 ± 0.20
	Day 8	1.20 ± 0.11	1.50 ± 0.21	1.57 ± 0.21	0.63 ± 0.17 *	0.40 ± 0.20 *	0.50 ± 0.22 *	0.50 ± 0.17 *
Collagen-I	Day 2	0.67 ± 0.21	0.50 ± 0.22	0.50 ± 0.22	0.83 ± 0.11	1.25 ± 0.17	1.25 ± 0.17	1.33 ± 0.21
	Day 8	1.17 ± 0.25	1.17 ± 0.17	1.33 ± 0.17	1.50 ± 0.18	2.00 ± 0.17	2.10 ± 0.18	2.00 ± 0.27
